# Reflections on Conducting a Large Replication Project in Sports and Exercise Science

**DOI:** 10.1007/s40279-025-02200-x

**Published:** 2025-06-16

**Authors:** Jennifer Murphy, Aaron R. Caldwell, Joe P. Warne

**Affiliations:** 1 School of Biological, Health and Sport Sciences, Technological University Dublin, Tallaght Campus, Blessington Road, Dublin 9, Ireland; 2 Institute for Community Health Innovation, University of Arkansas for Medical Sciences Northwest, Springdale, AR USA

## Abstract

This review reflects on the lessons and limitations of the first large, collaborative replication project in sports and exercise science. We discuss the challenges and barriers faced, while also exploring the broader contribution of replication to the field. This project faced many practical challenges when preparing studies for replication, specifically the poor reporting of statistical information, the availability of original raw data and the prioritisation of feasibility at the risk of some bias. However, we believe these issues reflect the larger sports and exercise science field. Therefore, our research culture needs to change to minimise the active engagement in behaviours that reduce reproducibility and replicability, and enable collective evaluation of research in line with the foundations of scientific rigour. In addition, discourse with the original study authors was a challenging process as many were unwilling to engage and this indicates a problematic perception of replication. We also reflect on the contribution of replication to theory development in sports and exercise science so that this review can serve as a valuable resource for understanding replication and can aid future replication efforts.

## Key Points

**Table:** 

Practical challenges to undertaking replication research include the poor reporting of statistical information and the availability of original raw data.	
Sociological challenges include discourse with the original study authors and the current incentive system that does not reward replication research.	

## Introduction

Some singular replications have been published in sports and exercise science but there was previously no large examination of the rate of replication in the field despite ongoing concerns about the replicability and verification of published effects [1, 2]. The Sports Science Replication Centre therefore aimed to provide an initial replication estimate of sports and exercise science research. To accomplish this aim, the Sports Science Replication Centre established a worldwide, collaborative laboratory network to undertake replication studies of applied sports and exercise science research, published between 2016 and 2021 in quartile 1 journals. Towards this effort, the protocol for selecting studies to be replicated was published, which provides a transparent description of inclusion and exclusion criteria [3]. Data collection was undertaken by collaborators in 10 countries and 29 replication studies were completed. We analysed 25 replication studies, and our overall findings were that 56% had the same null hypothesis significance testing outcomes as the original studies, using *p*-values as the criterion, and 36% of replication and original effect size estimates were compatible. Overall, 28% of the replications were deemed successful as they achieved statistical significance and compatible effect size estimates, assessed using a z-test.

Large replication projects such as the Reproducibility Project: Psychology [4], the Reproducibility Project: Cancer Biology [5] and Many Labs [6] have discussed the challenges they faced while undertaking their research. The selection of studies for replication is nearly always dependent on resources and prioritises feasibility for replicators. Studies in the Many Labs project were selected based on suitability for online data collection and a short data collection period. This therefore limited the generalisability of the conclusions to approximate replicability of psychology. The Reproducibility Project: Psychology openly stated that feasibility from both financial and time resource perspectives was a huge barrier to replication research. They acknowledged that original studies that were most resource intensive were not likely to be replicated. A major self-criticism of the Many Labs project was that study selection was subject to bias. The project leader, Ebersole, claimed replication teams were incentivised to select effects that would replicate rather than effects that would not [6, 7]. In addition, they believed that incentives were a barrier to participating in the project as participation was voluntary and required larger sample sizes than average to fulfil their high statistical power criteria [4]. Ultimately, resources and incentives are a huge barrier to undertaking replication research.

Large replication projects face common challenges such as resource limitations, statistical information gaps and a lack of incentives. The Sports Science Replication Centre’s replication project in sports and exercise science, the first of its type in our field, encountered similar obstacles. However, these challenges offer valuable lessons for future endeavours and provide insights into researcher attitudes towards replication. Replication projects, like ours, can expose transparency issues within the field and highlight areas for improvement. This review aims to reflect on our experiences and discuss the broader contribution of replication to sports and exercise science.

## Part I: Reflections on the Challenges in this Replication Project

### Barriers and Practical Challenges

The practical challenges we faced when preparing studies for replication included the reporting of statistical information and the availability of original raw data, and feasibility. We discuss these challenges in more detail in the following section.

#### Reporting of Statistical Information in Original Studies

A key concern about sports and exercise science research over the last number of years is the poor reporting of statistical information, particularly the reporting of the *p*-value alone [8, 9]. The availability of statistical information is directly related to the ability to closely replicate a study; not only are effect size estimates, means, variance, correlation values (for paired *t*-tests), exact *p*-values and test statistics needed for power analyses, but they are also essential for assessing replication outcomes. In a hypothetical world, if all of the statistical information was available from the original study (or at least the ability to calculate them), one could accurately compare mean differences, variances and the difference in effect size magnitudes. However, when this information is unavailable, comparisons become less accurate as they are based on conservative estimates, for example, relative *p*-values.

In our replication project, we estimated 36% of test statistics and 48% of degrees of freedom. We calculated 12% of the original effect size estimates using the original data and estimated 20%. This impacted our replication outcome assessments as it means that comparisons are less accurate when they are based on conservative estimates rather than true values. Similar issues were encountered by the Reproducibility Project: Cancer Biology, which aimed to replicate 193 experiments from 53 papers but were only able to repeat 50 experiments from 23 papers. This was because of the difficulty in obtaining statistical information, raw data and methodological clarifications [5]. Essentially, these challenges demonstrate the impact of open data and open communication with original authors. The continuation of “closed” science in sports and exercise science drastically decreased the ability to replicate effects exactly and hindered our attempts to estimate the replicability of the field.

One of the main analyses we used to evaluate the replication outcome in our project was the comparison of original and replication effect size estimates rather than relying on “vote-counting” (whether replications reached statistical significance). Even though effect size estimates should be reported in all research studies as “the primary product of research is one or more measures of effect size, not *p*-values …” [10], the reporting of original effect size estimates was a significant challenge for the replication project. In nine of the original studies that conducted paired *t-*tests, we calculated the Cohen’s *d*_*z*_ because six studies reported a Cohen’s *d*_*av*_, two studies did not report an effect size estimate and one study reported a Cohen’s *d*_*s*_. We could not also reproduce the original effect size estimate reported alongside the independent *t*-test selected for replication. The standardised mean difference, or Cohen’s *d* type effect size, is based on the difference between observations (mean difference), divided by some formulation of the standard deviation. The key difference between Cohen’s *d*_*av*_ and Cohen’s *d*_z_ is the calculation of the denominator; Cohen’s *d*_av_ uses the averaged standard deviation and Cohen’s *d*_z_ uses the standard deviation of the difference [11]. Lastly, we could not reproduce the partial eta squared in three of the analyses of variance while three additional analyses of variance did not report any effect size. Therefore, estimating the appropriate effect sizes certainly affected the overall outcome by reducing the accuracy of the replication rate.

Not only did the lack of reporting, or incorrect reporting, of effect sizes cause a barrier to assessing replication outcomes, but it also caused issues earlier in the planning process as the original effect size estimates were used to inform power analyses for the replication studies. In some cases, we had to conservatively estimate the effect size because test statistics were missing and/or only a relative *p*-value was reported. The use of conservative estimates of effect sizes decreased the accuracy of statistical comparisons between original and replication studies, thereby the initial estimate of the replicability of the sports and exercise science field. However, we could not exclude potential studies because they did not report full statistical information as this would tremendously decrease the number of studies available for replication. It would also bias the pool of studies for replication towards original studies that have fully reported all statistical information. The overall reporting rate of original effect size estimates was 68% in this replication project, which is lower than the 79% reported by Twomey et al. [12], albeit they had a much larger sample size of 300 studies. It is simply unacceptable to either not report or incorrectly report effect sizes in sports and exercise science when there is clear and helpful guidance on this topic [11, 13]. Additionally, this information is needed for meta-analyses to collate information on sports and exercise science topics but alarmingly, even these meta-analyses are not immune to basic statistical errors, [14] which provides a bleak outlook for the statistical competency of the field.

#### Bias Towards Resource Availability and Feasibility

This was one of the first replication efforts that attempted to semi-randomise the allocation of studies to collaborators, as this was a major risk of bias in prior efforts [15]. However, to allocate replication studies, feasibility had to be at the forefront of discussions and decisions particularly when all collaborators were completely voluntary. Feasibility naturally evoked a bias towards “easier to run” studies, which required an achievable number of participants to recruit, used less time-consuming or complex equipment, and had a shorter intervention period of less than 12 weeks. Unlike previous replication projects, we published a formalised selection protocol to enhance project transparency and minimise bias where possible [3]. Nevertheless, it was naturally impossible to eradicate bias.

Although the selection process was semi-systematic as we created an online survey to select studies, some of the decision making was still quite subjective, for example, we subjectively decided what we considered feasible or not. We gathered information during the collaborator recruitment process on what type (e.g. collegiate basketballers) and size of samples they typically recruited, and what the typical intervention length was for research studies in their laboratory. However, we still made feasibility assumptions on behalf of the project collaborators. Similarly, there was also bias from collaborators towards more feasible replication studies; when they were allocated a study, they were allowed to return the study with reasons about why it was not feasible for them, although there was no specific feasibility checklist in place. In summary, although we aimed to minimise bias, it was not possible to fully exclude bias from the process. Yet, we believe this is an improvement compared to other projects that did not transparently report their study selection process in full detail or allowed replicators to select the studies themselves and were subsequently criticised for it [15].

While the replication studies face many practical challenges in this project, these feasibility issues appear to plague all of sports and exercise science research; the use of small sample sizes has been an ongoing debate for some time in this field [16, 17], and there seems to be a trade-off between appropriately powering studies and the consideration of resource availability and feasibility. The result appears to be that feasibility is winning and we are rewarded with underpowered studies, inflated effect sizes and a questionably high positive results rate [12]. In combination with publication bias [8, 9], and the lack of transparency in reporting statistical information and providing raw data [18], we are left with a biased and non-replicable research record. Our research culture clearly needs to change; thus, this becomes a sociological challenge.

### Sociological Challenges

#### Incentives and Reward System

The practical challenges of undertaking research are well known and, as we have described, are no different for replication research. However, there is the added sociological challenge of conducting replication research as it relies on discourse with original authors and perhaps a lower incentive system. The incentive and reward systems that affect academia are certainly present in replication research, for example, publication for career progression [19]. There is a dilemma for open research advocates hoping to improve the quality of research; they want to contribute towards a project that aims to increase transparency in sports and exercise science, but it is also in human nature to seek a reward. As there was no reward system for this replication project (it was completely voluntary), the assumed incentives for taking part in this research were (1) intrinsic interest, (2) the experience of being involved with a large collaborative project or big team science [20] and, of course, (3) the traditional academic reward of publication. One hundred and eighty-nine sports and exercise science researchers expressed interest in the project, which clearly shows they are interested in improving research in the field or, at the very least, interested in learning how to conduct replication studies. Yet, 29 collaborators completed replications. This suggests that incentives are not always sufficient to overcome practical barriers in replication research.

There is an ongoing discussion of the workload creep of open science in comparison to closed science, where the only publicly available aspect of the research project is the final published article and not a transparent catalogue throughout [19]. Each replication study as part of this large project involved a power analysis, preregistration, study design and planning, communication with the original authors, a post-data collection report of the replication limitations and methodological differences, and open code and data. These were all in addition to the usual workload of research, for example, ethical approval, participant recruitment, data collection, data analysis and manuscript preparation. Therefore, there was a significantly high workload involved in the replication studies that may have deterred some interested collaborators. In addition to the view by some researchers that replication research is not creative, and the reluctance by journals to publish replication research [2], the sociological challenge of conducting this sports and exercise science replication project was high. This incentive system needs to change in the future to enable further large replication efforts.

#### Scientific Discourse and Open Communication/Original Author Response Rates

The replication project was a huge team effort including the collaborators (replicators), supervisory team, finance offices and ethics committees but one of the main sociological challenges was that it also needed input from the original authors. As per our selection protocol [3], ﻿we planned for our replication study methods to be as close as possible to the original methods; therefore, open communication with the original authors was essential. In addition, clarifications can be helpful and prevent any defensive backlash when an effect does not replicate in a subsequent study [21]. We contacted the original study authors to request raw data or full summary statistics for the key dependent variable to be replicated while explaining the purpose of our research. These authors were contacted when the studies entered the replication study pool after our selection process [3]. The corresponding authors were e-mailed and, if there was no response, we then followed up with the senior author on the paper (if they were not the corresponding author). If there was no response at all at this timepoint, the authors were not contacted again until the study was allocated to a collaborator for replication. The original authors were explicitly told at this stage that their study would be replicated regardless of a response. Of the 25 analysed replications, only 7 (28%) shared their raw data.

Each replication study was preregistered on the *Open Science Framework* (https://doi.org/10.17605/OSF.IO/3VUFG). These preregistrations followed the “Replication Recipe” template proposed by Brandt et al., [22] which asks the replicators to specify any deviations from the original study methods, sample size justification and any expected differences that could impact the replication outcome. However, before the preregistration was uploaded to the *Open Science Framework* project page, the preregistrations of each replication study were emailed to the original authors for review. We specifically asked the original authors to inform us whether they had comments or concerns on the replication study methods, or if they endorsed the methods proposed. Therefore, the replication preregistration was made available for critique to minimise random error and alternative explanations for replication outcomes [3, 23]. In total, 29 preregistrations were sent to the original authors of the selected replication studies and a timeframe of 2 weeks was given for the review deadline. Depending on the original author’s response, they were coded as “approved”, “concerns or comments made” (where further details could be provided in a free-text box) or “not reviewed”. Of the 29 preregistrations, 17% were approved, 14% had comments (e.g. “Will the force platform be the same make and model?”) and 69% were not reviewed at all. Of the 14 original authors contacted for methodological queries, 64% responded while 36% did not.

The low data sharing and response rates presented above hindered this project’s replicability, mirroring challenges faced by other replication projects. The Reproducibility Project: Cancer Biology outlined their difficulties in obtaining data from original authors because of poor response rates; the raw data share rate was 16%, 14% shared summary data and 68% did not share anything with replicators [24]. As detailed in an earlier section, the availability of raw data can lead to more accurate power analyses and evaluation of replication outcomes via the comparison of means, variances and effect size estimates. However, the level of communication from the original authors is also quite informative about the perception of replication in sports and exercise science. There were many concerns and gatekeeping from original authors who were naturally defensive about their work, did not want their work criticised, felt like it was a reputational attack and were generally unwilling to engage with replicators, perhaps owing to concerns over a potential failure to replicate their work. Throughout email communication with the original authors, this replication project was described as a “witch hunt”, “out to get people”, and a waste of time and resources. One original author also described why we should not replicate their work and provided us with the name of another researcher in the field whose work we should replicate instead. The challenging nature of the discourse with original authors indicates a problematic perception of replication, specifically replication failures, as surveyed sports and exercise scientists believe there is a negative stigma around non-replicable results [2]. Presumably, many of the original authors we contacted believed that if we failed to replicate their findings, it would invite criticism, and they were at fault somehow. However, there are other reasons for replication failure that are not related to expertise or original study error, and require an understanding of theory development. Specifically, replication can provide ongoing evidence to support original findings when results are compatible between two studies and provide evidence against them when results are not compatible, therefore, researchers should consider the total evidence for effects rather than focusing on stand-alone results for theory development. However, this does not appear to be common practice as there seems to be cultural inertia; failed replications do not seem to affect the citation patterns of original findings, and the replication result then goes unacknowledged [25].

#### Responding to Criticism

The attitudes towards the replication and re-evaluation of original findings illustrate the importance of respectful communication with original authors and the need to provide full clarity of the objectives of the replication research, particularly when science and the self cannot be separated [26]. Therefore, from the outset of this project, we prioritised respectful communication with the original authors and provided them with full details of the project and our intentions, answered all follow-up questions, shared the selection protocol with them to show that we did not cherry-pick studies to replicate and involved them in reviewing the preregistrations. Afterwards, we gave them an opportunity to review any manuscripts that were written up for individual publication. Furthermore, unless the direct actions of the original author were a barrier to replicating a study, for example, not providing raw data, we made any criticism of the work impersonal and focused on the ideas [27]. Until there is a better understanding of replication, and it becomes more mainstream in sports and exercise science, perhaps there will always be some reluctance or hesitancy from original authors to engage with replicators.

### Summary

In summary, this replication project faced both practical challenges, such as poor reporting of basic statistical information and data availability, and sociological challenges, notably the interactions between original authors and replicators. The practical challenges are commonly discussed issues in sports and exercise science and our results support previous findings that sports and exercise scientists actively engage in behaviours that reduce the reproducibility and replicability of our field, for example, a willingness to provide data or report full statistical information [1, 16, 18]. However, the sociological challenges of attempting to replicate studies are less commonly discussed. While methodological reform is crucial, addressing these issues alone neglects the underlying social dynamics hindering replication and indeed science as a whole, which is that scientific endeavour is a collective effort at improving knowledge in the long term [28]. To achieve a more transparent and replicable future for sports and exercise science, original authors and replicators must collaborate to prioritise the advancement of ideas and theories over individual interests. Otherwise, as evidenced by our project, we risk impeding progress by focusing on personal stakes rather than collective scientific goals.

## Part II: Reflections on Replication and its Contribution Towards Sports and Exercise Science

The first part of this review detailed the practical and sociological challenges faced in our large-scale replication project in sports and exercise science, which revealed a low replication rate of 28%. However, placing the responsibility for possible replication issues on methodological and statistical problems is neglectful of broader philosophical issues in our field [29]. As evidenced by some original authors’ reactions, there seems to be a misunderstanding of replication’s purpose and interpretation. Therefore, the second part of this review delves into the concept of replication itself, exploring its broader significance and contribution to the scientific process within sports and exercise science.

### Distinguishing Close and Conceptual Replications

Within sports and exercise science, the inherent complexity of human behaviour and systems complicates replication efforts. It becomes challenging to determine the precise conditions necessary to replicate prior findings because of the numerous differences in study designs and contexts [21]. As a result, researchers could then disagree over what constitutes a replication, and it can be challenging to attempt to closely replicate a theoretical concept from original studies [30]. Our project aimed to conduct close replications of the original studies where “ideally the only differences between the two are the inevitable ones” [22]. Consequently, we attempted to follow the original methods as exactly as possible including participant recruitment, sample characteristics, experimental set-up, instructions, measures and stimuli, and statistical analyses. While some original authors might believe their published methods are sufficiently detailed for replication, the lack of specificity often posed a significant challenge, particularly when authors were unresponsive. As discussed above, we contacted 14 original authors for further methods details. This helps to identify any confounding factors that could affect the outcome of the replication and prevent fruitless discussions on methodological deviations post hoc. While nine responded, with varying degrees of helpfulness, we were sometimes forced to proceed based on our best judgement and expertise. This highlights the crucial need for greater transparency and clarity in reporting research methods to facilitate successful replication endeavours, which were often found lacking.

On reflection of our project, it would be too simplistic of us to label all the replication studies as “close”. Perhaps some would be more aptly described as conceptual replications that have a “purposeful deviation” from the original study methods [30]. The distinction between close and conceptual replication became blurred at times. It was difficult to know if we had sufficiently included all the crucial elements that underpinned the original study and did not deviate from them. For example, we expanded the inclusion criteria for participants in one study by increasing the age range by 5–10 years, which increased the variance. In another study, the original study inclusion criterion was female volleyball athletes, but active female individuals who took part in any team sport were recruited in the replication study. Although these deviations inform us about the generalisability or boundary conditions of the effects under investigation, they might not be considered close replications. As one “cannot step in the same river twice”, so too will there inevitably be some differences between the replication study and the original study [21, 22]. The differences between the replication and original study also extend beyond the sampling variation [31]; therefore, it becomes crucial to assess whether the replication encompasses all theoretically relevant dimensions of the original study and contributes evidence for or against the effect in question. Additionally, it is argued that the procedural definitions for replication are irrelevant because the role of replication is to advance knowledge [21]. Our project aimed to replicate published studies faithfully, adhering to the original methods without modification or potential methodological improvement, thus fulfilling our aim of estimating replicability [3]. However, for future replications seeking to advance knowledge within sport and exercise science, we believe a deeper consideration of the theoretical underpinnings of the original study is essential and we encourage more specific single replication efforts in the future.

### Replication Quality

We reported difficulty in distinguishing between close and conceptual replication in our project, but the quality of these replication studies also needs to be discussed for full transparency. A replication study does not automatically mean that it is a high-quality scientific output. Although we stated in our proposal for selecting research to replicate that we would not aim to improve any study methods, replicating an original study with a poorly developed theory and poorly designed methods results in a poorly designed replication study. Some may understandably criticise this approach as being wasteful of already scarce research resources, but as per our aim, our approach was to take published studies at face value to see what we could learn.

We subjectively graded the replication quality across our studies as “poor”, “moderate” and “good” depending on the closeness of the sample characteristics, equipment and experimental set-up. Of the 25 studies, we graded 12 studies as “good”, 10 studies as “moderate” and 3 studies as “poor”. As one example, in the replication study of “Effect of different loading conditions on running mechanics at different velocities” (https://doi.org/10.17605/OSF.IO/C5ZP3), the treadmill sides were slightly raised above the belt and the Optogait, which was used to measure spatiotemporal variables, and did not always read the measurements at slower running speeds. Consequently, participants were encouraged to “lift their feet”, altering their leg length, which affected the key dependent variable, leg stiffness, as a result. Online images of the treadmill in the original study appear to carry the same limitation, but no issues were reported. We aimed to be transparent about the replication quality of the studies and reported any limitations and methodological differences and made them fully available online (https://doi.org/10.17605/OSF.IO/3VUFG). Therefore, even though we subjectively graded the replication quality, we encourage others to form their own judgements on the studies by reading the supplementary materials. If concerns arise regarding the quality of these replications, we must also question how the original research was published. If a study’s methods, theory or transparency were inadequate for replication, its publication raises doubts about the robustness and broader relevance of its findings. As Crüwell [32] suggests, if effects are so fragile that they cannot be replicated based on the published methods, their value is questionable, particularly in a field that does not emphasise self-correction where these issues are less likely to be identified.

Based on our difficulty in closely replicating studies at times, we believe that the original study authors have some responsibility for the lack of published information in manuscripts. Often, nuanced decisions made during the experimental design phase are under-emphasised in the published manuscript, creating engaging narratives but sacrificing scientific rigour and transparency. This makes the manuscript interesting but also incompatible with the scientific method and transparency [32]. While the replication teams share some responsibility for replication quality, the lack of transparency in original studies contributes to a low replication rate and allows for replicator degrees of freedom [33]. We have provided transparent access to our empirical decisions through supplementary materials, but this is of limited value when comparisons are hindered by the ambiguity of original studies. Therefore, we encourage all sport and exercise science researchers to include more methodological detail in their manuscripts and to use online repositories to supplement the manuscript with greater details on the methods, statistical practices and data. Ultimately, the lack of detailed information in methods and statistical analyses remains a root cause of many issues in sports and exercise science, making replication unnecessarily challenging.

## Part III: Future Implications

Having reflected on the challenges of our replication project and the broader role of replication in sports and exercise science, we now turn our attention to the future. In this final section, we examine the current trajectory of research in our field given prevailing practices and behaviours and propose ideals to strive for in order to enhance transparency and restore confidence in future findings.

### Replication and Theory Development: The Current Projection

Beyond discovering new ideas, science’s purpose lies in detecting errors and falsehoods to refine our imperfect understanding of the universe [34, 35]. A cornerstone of modern science is the ability to critically evaluate claims, rather than blindly accepting them [36]*.* However, as discussed in part one of this review on incentives, replication studies are not favoured in sports and exercise science and “research rewards accrue to those doing novel studies” [2]. Consequently, replication is not at the forefront of discussions for many sports and exercise scientists despite its contribution to theory development.

Many researchers in our field feel pressure to publish and we previously discussed how we have a crisis of incentives on our hands [2]. Like other fields, sports and exercise scientists are incentivised to engage in poor research practices to produce publishable findings [8, 9]. This is evident because of the high positive results rate (81%) and publication bias, indicating that some questionable research practices are occurring in the field, for example, HARKing (hypothesising after results are known) and *p*-hacking [8, 12]. The adoption of poor scientific behaviours, for example, potential questionable research practices [37] and an unwillingness to share data [18], lead to an excess of significant findings [12] that are potentially not replicable [1]. Given the current projection in the field, we should continue to expect a low replication rate, especially as our theory and hypothesis development in sports and exercise science is underdeveloped.

Overall, the open science reform movement has tended not to trace the roots of the replication crisis to the theoretical and philosophical underpinnings of our science [29]. Yet, replication is essential for theory development in sports and exercise science by allowing researchers to test the validity of scientific findings, as a single discovery is not sufficient to establish the validity of that discovery [38]. Ideally, we should be able to identify findings that are worth re-evaluating (i.e. failures to replicate) or corroborate findings with replications (i.e. successful replications), but this is not the norm. Despite the reliance on null hypothesis significance testing, many studies in sports and exercise science fail to formulate clear testable hypotheses [12], highlighting a lack of understanding of experimental design and statistical practices. When hypotheses are stated, they are frequently so vague that virtually any result could be used to support them [12]. Some researchers do not even state a hypothesis and continue to use null hypothesis significance testing anyway, or in contrast, authors state a hypothesis but conduct the study in line with an exploratory design, with many variables and statistical tests. This problematic approach can lead to an artificially high number of significant findings, particularly when hypotheses are not specified in advance, when researchers cherry-pick from multiple statistical tests, or when hypotheses are too vague to be falsified. Such findings are inherently difficult to replicate. This raises a crucial question: if researchers are not properly developing and defining their hypotheses, how can they situate their work within broader theoretical frameworks? We strongly advocate for more rigorous hypothesis development in sports and exercise science, and recommend Scheel et al. [40] for guidance. While awareness of these issues exists [2], the field continues to produce a plethora of unfalsifiable “positive” findings, driven by demands for novelty and publications. This leaves us with a research record that is increasingly difficult to trust. The question remains: how do we navigate this predicament and forge a path toward a more reliable and trustworthy body of knowledge in sports and exercise science?

### Managing Our Research Expectations

Should we persist in building theories upon studies with small sample sizes, noisy measurements and questionable statistics, the plausibility of entire research lines within sports and exercise science becomes dubious [41]. Such findings are of little use to researchers or practitioners. We can begin to address the replication problem in sports and exercise science by managing our research expectations and setting more realistic research goals. In line with Popperian ideas, Holtz [42] argued that the replication crisis in psychology could be attributed to epistemological deficiencies because researchers are “too eager to find their theories corroborated by empirical evidence, they do not consider competing theories often enough, and they often do not pay enough attention to the inferences that can be drawn from not finding the expected results”. A similar perspective could be applied to sports and exercise science. We must shift our focus towards theory development in terms of long-run probability, emphasising the accumulation of evidence based on sound methodological and statistical practices.

In this regard, setting realistic research goals necessitates a meticulous approach, including detailed research question development, a thorough review of relevant evidence and careful consideration of the target population for generalisation. It is far more valuable to rigorously test a simple question than to inadequately address a complex one, as the latter merely squanders resources. It is crucial to acknowledge that empirical data cannot definitively “answer” a research question nor prove a theory true. Instead, we can only corroborate theories through repeated testing, such as replication [43]. Unfortunately, the concept of falsifiability is often overlooked in hypothesis testing within sports and exercise science, leading to vague hypotheses and ambiguous findings. This, in turn, renders non-replications difficult to interpret, hindering the advancement of knowledge.

The low replication rate in our field will be debated in the future because non-replications will be dismissed on the grounds that methodological details were varied, even though the original theory did not specify the importance of these details [40]. To improve our future theory testing in sports and exercise science, researchers should specify how their independent and dependent variables will be operationalised, how many participants they will collect data on, which exclusion criteria they will apply, which statistical method they will use, and how to decide whether the hypothesis was corroborated or falsified [40]. Lastly, we also encourage the use of exploratory testing to formulate better research ideas rather than immediately using confirmatory testing and neglecting the groundwork of developing a research question [40].

### An Educational Overhaul

Our previous survey on reproducibility and replicability found that statistical education was a key area of concern for researchers in the field [2] and we have acknowledged this issue throughout this review. We previously stated that ﻿the “sports and exercise science field will reap the reward of an investment in better statistical education in the future” [2] but perhaps this was too simplistic, and an overall research methods educational overhaul is needed. Research methods and statistics education in our field often adopt a rigid black-and-white approach, focusing on absolutes: confirmatory versus exploratory research, specific tests for specific data types and a binary interpretation of *p-*values. However, this neglects the nuanced “grey areas” inherent in research, and rarely do educational courses offer a philosophical approach to theory development. Uncertainty is an unavoidable aspect of research, whether in individual predictions or broader patterns. Embracing this uncertainty, as Ting and Greenland [44] suggest, is crucial for fostering a more mature and realistic understanding of research; this should be reflected in how we write our manuscripts and discuss the results of single studies. Limitations sections, which are often relegated as afterthoughts in publications, should be recognised as central components of research, as they are crucial for assessing the generalisability of findings. We must resist the urge to overstate practical applications while downplaying limitations, accepting that many studies in sports and exercise science are simply too limited in scope to offer definitive conclusions [41].

## Conclusions

This review aimed to reflect on the challenges encountered during the first collaborative replication project in sports and exercise science, while also exploring the broader contribution of replication to the field. Prior research has highlighted the lack of transparency in reporting statistical information and sharing raw data as a prevalent issue, which is particularly exacerbated by publication bias. Our replication project reaffirmed this concern, as the lack of detailed information in original studies hindered the replication process. While original authors bear some responsibility for the low replication rate because of insufficient transparency and the resulting replicator degrees of freedom, replicators also faced challenges in maximising replication quality and ensuring close, rather than conceptual, replications. This underscores the intricate interplay between original research practices and the complexities inherent in replication endeavours.

In addition to the practical challenges in this project, there were also sociological challenges that hindered replication attempts. Open discourse with the original authors is an important step when replicating research but a large proportion of the original authors were not receptive to our project. Some even expressed hostility towards our replication attempts, a reaction we believe stems from a misunderstanding of replication’s purpose and the prevailing closed science culture. A potential future solution to replication opposition is educating researchers on the purpose of replication and its contribution to theory development. Furthermore, an increased educational emphasis on the collective development of theory, including the important aspects of theoretical falsification, should be adopted. A wider concern is the more embedded incentive structures in academia that may prove harder to change.

To rebuild trust in published sports and exercise science findings, we must shift our perspective and prioritise studies that demonstrate transparency and acknowledge their limitations. A study that openly discusses its limitations and expresses uncertainty (e.g. reports confidence intervals around effect size estimates) should be more trustworthy than one with vague hypotheses or overgeneralised conclusions. Furthermore, we must manage our research expectations, embrace uncertainty and normalise the rejection of research theories so that we appropriately state the practical applications of our work while highlighting any limitations. We hope that this thorough exploration of our large replication project’s challenges will serve as a valuable resource for future replication efforts, both within our field and beyond.

## Declarations

### Availability of Data and Material

Raw data and other supplementary materials are available at https://doi.org/10.17605/OSF.IO/SFBVA.

### Code Availability

The code is available at 10.17605/OSF.IO/SFBVA.
